# The role of Exosomes in the Pathogenesis of Nasopharyngeal Carcinoma and the involved Clinical Application

**DOI:** 10.7150/ijbs.59688

**Published:** 2021-05-27

**Authors:** Huidan Luo, Bin Yi

**Affiliations:** Department of Clinical Laboratory, Xiangya Hospital, Central South University, Changsha, Hunan Province 410008, China.

**Keywords:** exosomes, NPC, angiogenesis, tumor metastasis

## Abstract

Exosomes are nanoscale membrane vesicles, which carry biologically active substances of their cell of origin and play an important role in signal transduction and intercellular communication. At present, exosomes have been identified as a promising non-invasive liquid biopsy biomarker in the tissues and circulating blood of nasopharyngeal carcinoma (NPC) and found to participate in regulating pathophysiological process of the tumor. We here review recent insights gained into the molecular mechanisms of exosome-induced cell growth, angiogenesis, metastasis, immunosuppression, radiation resistance and chemotherapy resistance in the development and progression of NPC, as well as the clinical application of exosomes as diagnostic biomarkers and therapeutic agents. We also discuss the limitations and challenges in exosome application. We hope this review may provide some references for the use of exosomes in clinical intervention.

## Introduction

NPC is a squamous cell carcinoma arising from the epithelial cells of the nasopharyngeal mucosa. Epstein-Barr virus (EBV) infection is one of the essential factors leading to NPC 1,2. The geographic distribution of NPC is very skewed, mostly in southern China and southeast Asia, where NPC is still a major threat to people's life 3, despite the substantial improvements in large-scale screening and therapeutic strategies cut down the morbidity and mortality of the tumor 4. NPC is not usually detected at the early stage and patients with advanced NPC are frequently accompanied by lymph node infiltration and distant metastasis. Some patients even develop resistance during radiotherapy or chemotherapy, which may lead to a very bad outcome 5,6.

Exosomes are cell vesicles with a diameter of 40-100 nm, secreted by various types of cells outside the cell by fusing with the plasma membrane. The plasma membrane buds inward to form early endosomes, which then mature into late endosomes and multivesicular bodies (MVBs) with intraluminal vesicles. When late MVBs fuse with the plasma membrane, exosomes are released 7,8. The content of exosomes consists of a variety of substances including lipids, nucleic acids and proteins specifically associated with the plasma membrane and cytoplasm. Exosomes involved in lipid metabolism have been found in plasma, urine, semen, saliva, etc. 9-11. Exosomes can act as messengers to mediate cell communication and deliver the constituents to the recipient cells to perform crucial roles 12-14. The uptake of exosomes is not random, but dependent upon the interaction between the protein on the surface of the exosomes and the recipient cells. Currently, exosomes are basically isolated by using ultra-high-speed centrifugation; other methods include size-based separation, polymer precipitation, immunoaffinity capture and the recent emergence of microphallus derivative technology 15. Corresponding kits for exosome extraction have also been developed in recent years. Ultra-high-speed centrifugation technology has been known as the 'gold standard' for exosome isolation. The isolated exosomes are usually observed by transmission electron microscopy. Nano-tracking analysis is adopted to measure their size and western blotting is performed to trace the marker proteins such as CD9, CD63 or CD81 16.

The poor prognosis of NPC is mainly attributed to insufficient consciousness of the prominent symptoms of the disease, the limited detections and therapeutic options 17. In order to improve the prognosis of the patients, it is necessary to better understand the pathogenesis of NPC and to develop new therapeutic targets and effective strategies. Exosomes are of great significance in the occurrence and progression of NPC, which can be classified into EBV-related exosomes, exosomes derived from NPC cells or mesenchymal stem cells, and other types of exosomes 18. The exosomal components enter the recipient cell through the membrane fusion with the target cell, and participate in many important physiological and pathological processes 19-23. Originating from extracellular vesicles, exosomes have a unique source of cargo, capable of reproducing the molecular characteristics of parent cells in the nucleus 24. There is growing evidence that different types of exosomes target their respective receptors to exert a variety of biological effects on NPC cell proliferation and function, resulting in promoting or suppressing the tumor growth 18,25-27. In addition to serve as intercellular carriers for material transfer, exosomes can be utilized as biomarkers for disease diagnosis 28,29. Exploring the role of exosomal molecules in the progression of NPC will facilitate to develop new biomarkers for the early diagnosis of NPC, as well as to provide novel treatment ideas to improve the therapeutic efficacy.

## Exosomes affect various processes of NPC

Exosomes related to NPC have definite significance for the occurrence and progression of NPC. Exosomes carry cancer-aggravating or oppressing molecules that act on NPC or other stromal cells, which exert influence on different stages of NPC development and progression.

### NPC-related exosomes promote cell growth

The onset of NPC is etiologically associated with EBV infection. The establishment of EBV latent infection in pre-invasive nasopharyngeal epithelium is considered to be an early stage of NPC pathogenesis. A mass of viral products are expressed during the latent phase of infection, including EB virus nuclear antigen (EBNA) 1, EB virus-encoded latent membrane proteins (LMP) 1 and 2, EB virus-encoded RNA (EBER), BamH Ⅰ A rightward transcripts (BART), and some incubation period mRNA, with which EBV-related exosomes coexist 30,31. LMP1 is predominantly produced in EBV infection, closely linked to the occurrence and growth of NPC. It has been shown to have multiple functions *in vitro*, covering promoting cell growth, protecting cells from apoptosis and enhancing cell motility 32. LMP1 packaged by exosomes can activate normal fibroblasts and turn them into cancer-related fibroblasts through the key signaling pathway of nuclear factor (NF)-ƙB p65 33. Studies have revealed that LMP1 in NPC exosomes upregulates syndecan-2 (SDC2) and synaptotagmin-like-4 (SYTL4) via NF-κB signaling to stimulate the secretion of extracellular vesicles (EVs), promotes cell proliferation and tumor growth by activating ERK and AKT signal pathways and inducing vascular endothelial growth factor (VEGF) receptor expression 34,35. BART1 miRNAs are thought to negatively regulate LMP1 expression, which may be in favor of NPC pathogenesis 32. LMP2 can integrate into exosomes and then be released into the recipient cells 26. In NPC patients, the expression of miR-24-3p, miR-891a, miR-106a-5p, miR-20a-5p and miR-1908 in sera and exosomes is significantly different from that in the healthy controls. These miRNAs have impacts on NPC cell proliferation and differentiation via downregulating MARK1 signal pathway 36. Exosomes secreted by tumor cells and stromal cells are the key mediators of cell-to-cell communication in the tumor microenvironment, which facilitate tumor evolution and benefit other aspects of NPC aggravation 37.

### NPC-related exosomes mediate angiogenesis

**Figure 1:**
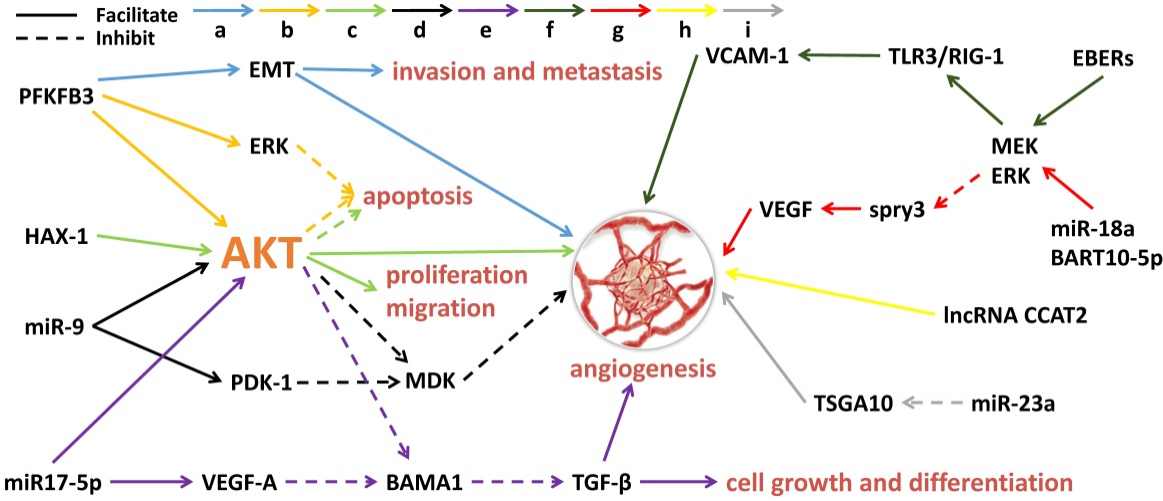
** Exosomal molecules and their pathways involved in angiogenesis of NPC.** 'a' to 'i' represents nine different molecular pathways. Multi-factors and pathways affect NPC angiogenesis, including some miRNAs, virus-related RNAs and proteins. The solid line indicates that the molecule can encourage the downstream signaling and the dotted line indicates inhibition.

Angiogenesis is the process by which new blood vessels form. The formation of tumor blood vessels can enhance tumor growth, invasiveness and metastasis, leading to poor prognosis 38. Tumor angiogenesis actually starts with tumor cells releasing molecules that send signals to promote blood vessel growth, causing local imbalances between factors that stimulate and inhibit angiogenesis 39. The major contributors to tumor angiogenesis include the angiopoietin (Ang)/Tie-2(Tek) pathway, the VEGF family and its receptors. Mechanistically, angiogenesis is a critical event in NPC metastasis. Growing evidence suggests that exosomes derived from NPC can fine-tune endothelial cell characteristics to facilitate angiogenesis, especially under hypoxic conditions 40 (**Figure 1**). Quantitative proteomic analysis have demonstrated that the expression of angiogenic proteins, ICAM-1 and CD44v5, is up-regulated in the exosomes of NPC, while the expression of angiostatin TSP-1 is down-regulated 41. As expected, these exosomes significantly contribute to the NPC angiogenesis. Some proteins for angiogenesis regulation are available in NPC tissues and exosomes. The glycolysis regulators of enzyme PFKFB3 and HS1-related protein X-1 (HAX1) can activate the ERK/AKT pathway and affect proliferation, migration, apoptosis and angiogenesis of the NPC cells 42,43. Similarly, a large number of miRNAs from NPC-derived exosomes have been proven to participate in tumor angiogenesis. MiR-23a targets and inhibits testis-specific gene antigen (TSGA10, an anti-angiogenic factor), resulting in angiogenesis in NPC 44. Highly expressed exosomal miR-17-5p encourages angiogenesis in NPC by targeting BAMB1 and regulating AKT/VEGF-A signaling 45. Long non-coding RNA (lncRNA) CCAT2 from NPC-derived exosomes can promote angiogenesis 46. EBV-related products in the exosomes of NPC can also affect tumor progression, such as the EBV-encoding RNAs (EBERs). For instance, EBERs regulate the expression of vascular cell adhesion molecule 1 (VCAM-1) through TLR3/RIG-I to induce angiogenesis 47; EBV-miR-BART10-5p and miR-18a strongly promote angiogenesis and tumor growth of NPC by mediating the expression of VEGF and HIF1-α in a Spry3-dependent manner 48. More important, the exosomal molecules not only have the ability to accelerate angiogenesis but also hold inhibitory functions. Juan Lu, et al. have pointed out that overexpressed exosomal miR-9 inhibits angiogenesis and metastasis of NPC via targeting a pro-angiogenic protein of MDK and regulating PDK/AKT signal routing 49.

### NPC-related exosomes promote epithelial-mesenchymal transition (EMT) and metastasis

NPC frequently metastasizes beyond the nasopharynx through additional blood vessels and lymph nodes, which is the most common reason for poor prognosis. EMT is a process by which the epithelial cells are transformed into quasi-mesenchymal cells. During this process, the interaction and interference between the cells and the extracellular matrix are reshaped with increased invasive and metastatic properties, resulting in separation of epithelial cells from each other and the basement membrane. Recent studies have revealed that EMT can enhance the mobility and invasiveness of the cell to gain greater metastatic potential, which prompts tumor metastasis and progression 50,51. Matrix metalloproteinase 13 (MMP-13) in NPC-related exosomes upregulates vimentin and reduces cadherin to facilitate tumor invasion, metastasis and angiogenesis; on the other hand, MMP13 expression is trans-activated by hypoxia-inducible factor α (HIF1α) 6,52. Exosomes can mediate continuous interference between cancer cells and stromal cells and the effect becomes stronger under hypoxic conditions 53. HIF1α is able to activate MMP13 and participate in cell metastasis itself, causing mutual change of EMT-related E/N cadherin expression 54. The level of endogenous HIF1α can be enhanced by LMP1 while exosomal HIF1α is supportive for the pro-invasive potential of LMP1-positive exosomes associated with NPC 54,55. The level of LMP1 is positively correlated with the expression of EMT markers and it functions to activate MMPs and miR-10b, inhibit miR-204, and consequently benefits the invasion and metastasis of NPC, whereas the invasion and metastasis process can be inhibited by miR-203. Moreover, LMP1 has been demonstrated to be able to stimulate the expression of miR-10b, which promotes cell migration and invasion via silencing HOXD10 and activating Twist 56. Analogous to LMP1, LMP2 can also induce EMT-like changes in NPC cells 57. The ERK1/2 pathway participates in raising the production of transcription factor Fra-1 and the level of MMP9; on the other hand, LMP2 and LMP1 phosphorylate 4EBP1 through the PI3K/Akt/mTOR signaling to elevate the expression of metastatic tumor antigen 1 (MTA1), thereby, expediting the invasiveness and metastatic ability of the NPC cells 2,58,59. Fibroblast growth factor (FGF) 19 in the exosomes of NPC mesenchymal stem cells can encourage occurrence, proliferation and metastasis of the tumor and the mechanism involves activating the FGF19-FGFR4-dependent ERK signal cascade and regulating EMT to stimulate the growth of NPC 60. PFKFB3 and HAX-1 can also strengthen the invasion and metastasis of NPC 42,43.

### NPC-related exosomes function in immune response

**Figure 2:**
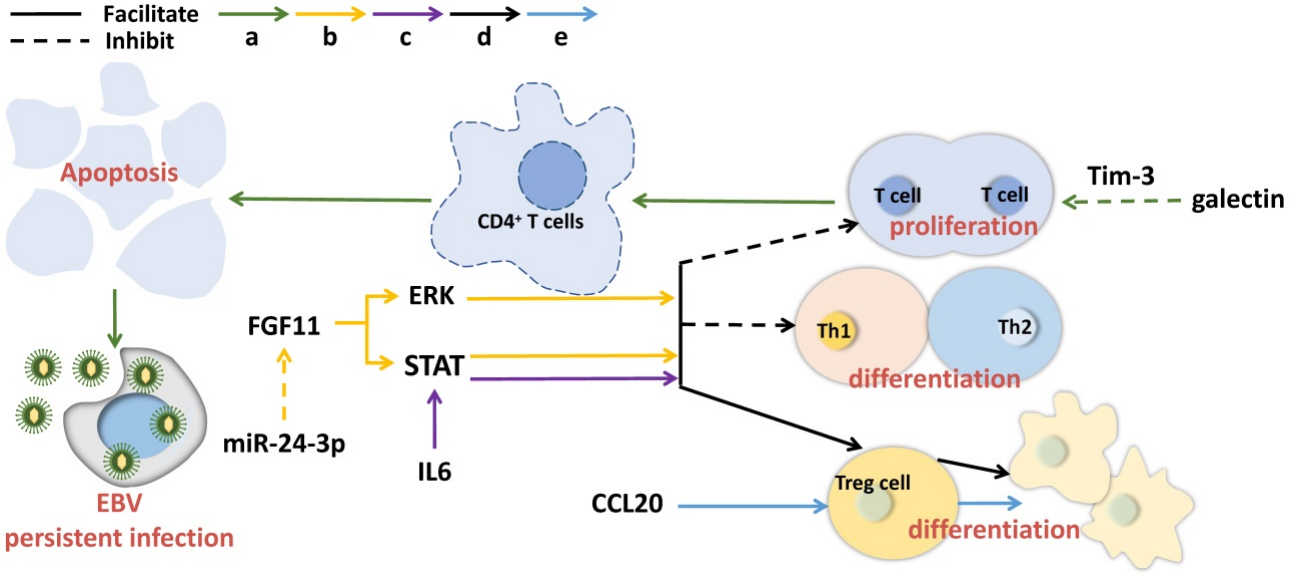
** Exosomal molecules and their pathways involved in immunosuppression in NPC.** Exosomal molecules can affect T cell activity, maintain continuous EBV infection and induce immunosuppression. The solid line indicates that the molecule can promote the downstream signaling and the dotted line represents inhibition.

The salient feature of NPC is the infiltration of a large number of non-malignant white blood cells in the primary tumor, mainly consisting of T lymphocytes and a small number of B cells, macrophages and dendritic cells, etc. However, this leukocyte infiltration has been found to disappear in the process of metastasis, replaced by rapidly and massively proliferated malignant cells with obvious anti-tumor immune feature 61. Exosome-derived cytokines and substances, such as galectin-9 and CCL20, can induce local accumulation of regulatory T cells (Tregs) and promote NPC aggressiveness 62. The immunomodulatory protein galactin-9 in EBV-infected NPC exosomes binds to ligand Tim-3, triggering apoptosis of mature CD4+ lymphocytes 63. LMP1 can provoke galectin-9 expression, resulting in the release of exosomes containing LMP1 and galectin-9, and the recombinant of LMP1 with galectin-9 can induce strong inhibition on T cell proliferation in contrast to galectin 9 without LMP1 synergy 64. Hypoxia is able to raise the level of miR-24-3p in NPC cells, sera and exosomes and enhance the inhibitory effects on T cell proliferation, Th1 and Th17 differentiation, as well as generate Tregs suppression via repressing FGF11 65. In addition, NPC exosomes have been observed to have the similar T cell inhibitory effect as the TW03 exosomes (EBV cell-negative or positive) do, by imposing on the pro-inflammatory cytokines 36. IL-6 is a growth factor for a great deal of tumors. In NPC, exosomes have been demonstrated to enable to improve the production of IL-6 from macrophages to promote tumorigenesis 66. As the core molecules that mediate immune suppression, Tregs can play tumor immune escape. It has been reported that exosomal chemokine CCL20 can recruit Tregs to tumor tissues, induce T cells to transform into inhibitory Tregs, and enhance Treg suppression 67. In addition, gamma herpesvirus infection can change exosome protein contents in B cells 68. Taking together, exosomal molecules can affect T cell activity, maintain continuous EBV infection and induce immunosuppression (**Figure 2**).

### NPC-related exosomes induce radiation resistance

Radiotherapy is the major treatment for NPC, but radiation resistance seems inevitable in this process in some patients. Treatment failure of local radiotherapy accompanied with local recurrence or distant metastasis continues to be a severe challenge for NPC management 69,70. Exosomes can arouse radioresistance by enhancing intercellular communication and cytotoxic damage in NPC cells 71. Some molecules originated from NPC-related exosomes have been confirmed to be radiosensitive. It has been found that CircMYC, a newly discovered circular RNA (circRNA) in the circulating exosomes of NPC patients, is radioresistant and supportive for cell proliferation, and high level of circMYC is correlated with NPC recurrence 25. Similarly, LMP1, as a vital cancer-promoting factor, exerts its carcinogenic effect by activating the P38 MARK signal of receptor cells to stimulate radiation resistance 72. Studies have shown that different cell lines of NPC have different radiation sensitivity, of them, CNE2 being the strongest. Distinct from other cell lines, the secretory proteins expressed in CNE2 cells are mainly transported through non-classical pathways and exosomes 73. In general, exosomes play a prominent role in transmitting radioresistance in NPC.

### NPC-related exosomes induce chemotherapy resistance

In lung cancer exosomes, abnormal expression of some miRNAs and mRNAs regarding to cisplatin (DDP) sensitivity has been discovered *in vitro*, accompanied by decreased sensitivity of the tumor cells to cisplatin, and exosomes can transfer this DDP resistance to untreated cells to induce resistance 74. NPC patients also develop resistance to drugs, where exosomes may be the transmitter. Doxorubicin-resistant endothelial cells have been demonstrated to facilitate aggression, metastasis, EMT and chemoresistance in NPC via exosomes 75. Overexpressed DDX53 has been detected in a variety of cancers and proven to be able to cause Taxol-resistance in cervix cancer cells *in vitro* through upregulating MDR1 76. Likewise, a latest study has reported that exosomes derived from Taxol-resistant NPC cells can transfer DDX53 resistance to the parental cells, while inhibiting the secretion of exosomes can block this process, thus revealing a new mechanism of drug resistance in NPC 77.

## NPC-related exosomes in clinical application

**Figure 3:**
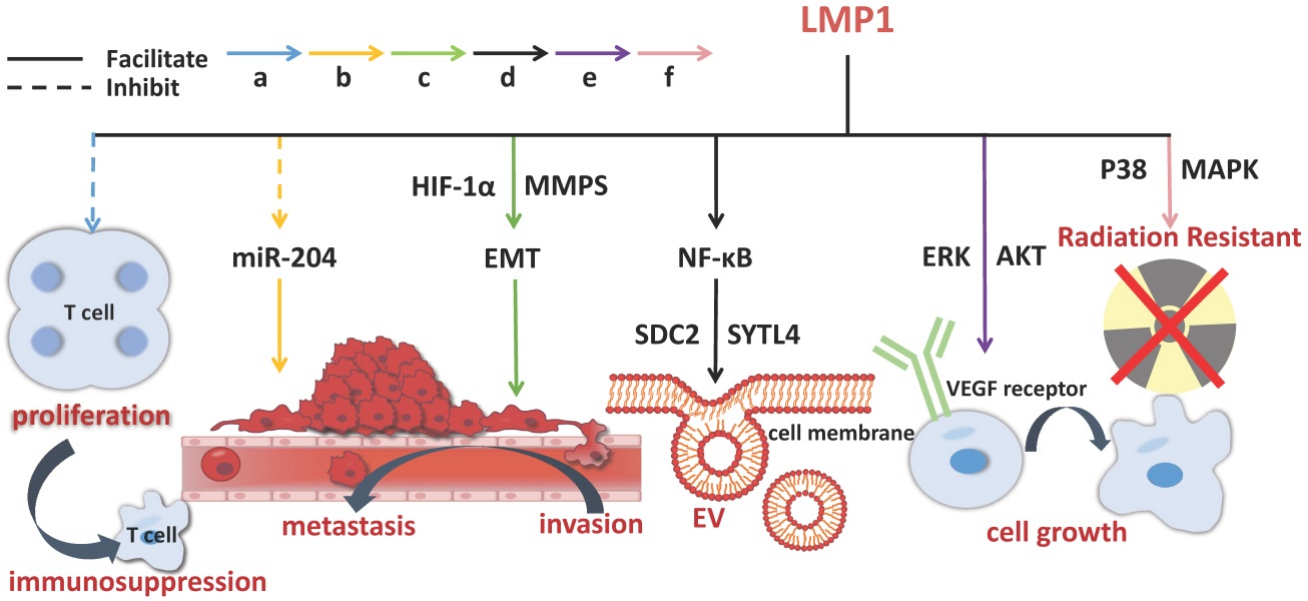
** The molecular mechanisms of exosomal LMP1 in NPC pathogenesis.** LMP1 promotes the progression of NPC through multiple channels, including stimulating EVs secretion, improving cell growth, prompting invasion, metastasis, and immunosuppression. The solid line indicates that the molecule can enhance the activity of its target and the dotted line indicates inhibition.

Some exosomal molecules have multiple functions in the progression of NPC, such as LMP1 (**Figure 3**), and have been widely used, especially in clinical application for NPC diagnosis and treatment.

### Exosomes applied in NPC diagnosis

Although the incidence of NPC has declined to a certain extent, its early detection is still a challenge due to its atypical symptoms and hidden location. Many NPC patients are already at an advanced stage at the time of diagnosis, worsening the outcome of the disease. Early diagnosis and interference are very important for NPC prognosis. Given that EBV is an influential issue for NPC, various EBV assays aiming at EBV-DNA and the relative antibody detection have been developed, but their sensitivity and specificity cannot satisfy the needs of the clinic 78,79. Hence, exploring novel biomarkers and methods for early NPC diagnosis becomes exigent. As a non-invasive test, liquid biopsy, capable of detecting tumor cells, tumor-derived nucleic acids and exosomes in the circulating body fluid of patients, is believed to be more practical than the traditional tumor biopsy. Currently, the application of exosomes in cancer diagnosis and surveillance has aroused extensive attention 80,81. The unique biogenesis of exosomes build up the ability to circulate freely in body fluids as various molecule carriers 82. Many contemporary studies emphasize that biomolecules in NPC exosomes can serve as novel diagnostic tumor biomarkers with the advanced technology platform focusing on nanoparticle identification. For example, Cytoflex, a new generation of flow cytometer equipped with 405 nm laser, has the ability to identify serum exosomes and microparticles 83,84. NanoSight is an updated instrument, which can directly detect as small as 10 nm nanoparticles/exosomes and give a size-distribution graph 49. EBV infection in NPC can induce differential expression of intracellular lncRNA in infected cells, exosomes and tumors, suggesting the potential clinical application of lncRNA as a biomarker 85. Plasma and exosomal BART miRNAs produced by EBV-infected NPC cells have been identified as new indicators of NPC 86. Exosomal circMYC has been proven to be correlative to radiation tolerance, and ROC analysis show that it has the potential to distinguish radiation tolerant NPC patients from the sensitive ones 25. Exosomal molecules can be tested coupled with the existing EBV detection. Detection of cyclophilin A (CYPA) in sera and exosomes combined with EBV-VCA-IgA and LMP1 has been employed to diagnose NPC, where CYPA in exosomes exhibits a much higher level than that in whole sera 87. In addition to be taken as biomarkers, exosomes have also been exploited to develop a type of exosomal nanovesicles *in vivo* as a contrast agent for H_2_O_2_-responsive catalytic photoacoustic imaging (PAI) in NPC, which achieves excellent lysosomal escape ability, strong stability and high sensitivity, better than the traditional non-biological materials for NPC detection 88. Notably, the molecules on exosome surface such as CD9, CD63 and CD81 have been used to label the exosome to detect its secretion for tumor monitoring 89.

### Exosomes applied in NPC treatment

Exosomes can provide protection for their contents against proteolytic digestion or drug action, which may be one of the causes for the failure of drug effect on NPC treatment, as exosomal cancer-promoting molecules move from the cancerous cells to the recipient cells. Increasingly, exosomes are being recognized as alternative therapeutics for NPC for their ability to induce potent cellular responses. For instance, targeting exosomal EBV-LMP1 transfer and miR-203 expression have positive significance for EBV-targeted therapy by aspirin in invasive NPC 90. The commonly used targets for tumor control include PD-1/PD-L1, VEGF and EGFR. Substances used for immune escape therapy such as PD-1 and CTLA-4 can activate cytotoxic T cells but that action is frequently blocked, compromising the therapeutic effect 91. Exosomes may offer solution to this issue because they have distinct advantages that uniquely position them as vehicles for the delivery of cancer drug when the molecules are included or linked to them with a ligand capable of accurately binding to their targets 92. In NPC, a number of key regulatory molecules involved in the tumor pathological process have been identified and a few of them have been used for novel cancer immunotherapy *in vitro* or *in vivo*. A research team has worked to incorporate antagomiR targeting BART10-5p and miR-18a (which are intended to promote angiogenesis) into iRGD-tagged exosomes, resulting in preferentially suppressed angiogenesis and growth of NPC 48. Delivering miR-34c (a tumor suppressor of NPC) to the cancerous cells through mesenchymal stem cell exosomes can impede β-chain protein and slow down the invasion, metastasis, proliferation and EMT of NPC cells, ultimately inhibiting tumor deterioration and radiation resistance 5.

### Limitations and challenges of exosomes in clinical applications

Although researches on NPC exosomes have achieved certain accomplishments, there are also challenges and limitations in the application process.

The first lies in the means of detection. Despite the fact that tumor exosomes are attractive biomarkers for tumor diagnosis, their early detection for clinical use is still challenging. Some researchers have established a creative exosome detection method with high sensitivity and specificity, which refers to recombinase polymerase amplification triggered by adjacent ligation and transcription-mediated amplification (PAL-RPA-TMA) 93; however, this approach requires innovative construction of DNA-labeled antibodies for specific detection targets (such as the proven biomarker LMP1). Based on hybrid chain reaction (HCR) and CRISPR-Cas12a double amplification technology, apta-HCR-CRISPR has been developed to directly examine extracellular vesicle proteins, which has attained high sensitivity 94. An immunosorbent analysis adopting micro fluidic drop technology has been exploited to quantitatively identify exosomes 95. Nonetheless, these detection methods are complicated, time consuming and costly, and more effective and feasible platform for exosome detection needs to be fulfilled.

The second involves the selective exosomal cargo loading mechanism driving biomolecules sorting into exosomes. Despite the entry of exosomes into recipient cells is non-specific, the exosome uptake mechanism depends on the recipient cells 96. Studies have shown that seven miRNAs (let-7b-5p, miR-140-3p, miR-144-3p, miR-17-5p, miR-20a-5p, miR-20b-5p and miR-205-5p) are consistently upregulated in the plasma of patients with NPC; however, the plasma-derived exosomes yield the opposite outcome, indicating that miRNAs in plasma-derived exosomes have different expression mechanism from those in plasma 97,98. The same phenomenon exists in the expression of viral miR-BART17 of EBV in NPC patients, where its concentration in plasma differs from that in plasma-derived exosomes 99. It has been revealed that one of the key mechanisms for LMP1 to enter exosomes correlates to the C-terminal farnesylation of UCH-L1; inhibition of UCH-L1 deubiquitination activity exerts an anti-invasive effect on metastatic cancer cells 100,101. CD63 has been found to be a specific protein on the surface of exosomes, which can bind to LMP1 and regulate LMP1 exosomal packaging 102,103. Understanding the specific exosomal loading mechanism of how the NPC-related molecules selectively enter the recipient cells and how exosomes make cargo selection may benefit developing alternative policy to improve NPC diagnosis and treatment.

A study comparing the effect of conventional doxorubicin chemotherapy with the replacement therapy of Ag-TiO2-catalyzed reactive oxygen species generation in the treatment of 5-8F NPC cells has pointed out that beyond the classic chemotherapeutic agent, Ag-TiO2 in the photo-catalytic process also exhibits cytostatic activity; tumor cell damage induced by cytostatic treatment enormously changes the number of released exosomes and leads to the predominance of tumor inhibitors in the exosomal miRNA profile 104. Different treatment option has different impact on NPC progression and the involved mechanisms may be varied. The current strategies for NPC include radiotherapy, adjuvant chemotherapy, concurrent chemoradiotherapy and induction chemotherapy. Radiotherapy is classified into intensity-modulated radiation therapy (IMRT), intensity-modulated proton therapy (IMPT) and intensity-modulated carbon therapy (IMCT). The commonly used agents for chemotherapy are cisplatin and fluorouracil 3. Probing the mechanisms of different therapeutic strategies and their effects on NPC-related exosomes will help break some new ground in dealing with NPC.

There are also limitations in exosome application. Due to the characteristics of stability, permeability, biocompatibility and extremely small size, exosomes can deliver drugs and nucleic acids as carriers 105,106, however, most of them stay in the theoretical stage, far from practical applications 107. The disadvantage of low drug loading rates and clinical production levels raise barriers for exosomes to target recipient cells with effectiveness and sufficiency. In addition, the non-tumor type specificity of exosomes in the body 108, and the protective effect of exosomes on their contents also restrain them to serve the clinic for precise tumor treatment.

## Conclusions

With the improvement of diagnostic methods and treatment strategies, the incidence and prognosis of NPC have been improved, however, the mechanism of the tumor's development and progression is unclear, and problems emerge in practical treatment. The exosomes secreted from different types of NPC cells contain a variety of biomolecules, which participate in the circulation of body fluids, and affect the growth, angiogenesis, metastasis, immune response, radiation and chemotherapy sensitivity of NPC. As a promising non-invasive liquid biopsy biomarker, exosomes are highlighted in developing new diagnostic and treatment methods of NPC, but there are challenges in their clinical application. Conclusively, this article reviews the molecular mechanisms of NPC-related exosome-induced tumor progression in NPC and the potential clinical application of exosomes, which we hope may provide some references for the use of exosomes in clinical intervention.

## Abbreviations

BART: BamHⅠ A rightward transcripts
CCL20: C-C chemokine ligand 20
CD44v5: CD44 variant 5
DDP: cis-diamminedichloroplatinum(II)
circRNA: circular RNA
CTLA-4: cytotoxic T lymphocyte associate protein-4
CYPA: cyclophilin A
EBER: Epstein-Barr virus-encoded RNA
EBNA: Epstein-Barr virus nuclear antigen
EBV: Epstein-Barr virus
EGFR: epidermal growth factor receptor
EMT: epithelial-mesenchymal transition
ERK: extracellular signal-regulated kinases
EV: extracellular vesicles
FGF: fibroblast growth factor
HAX-1: HS1-associated protein X-1
HCR: hybrid chain reaction
HIF1α: Hypoxia-inducible factor α
lncRNA: long non-coding RNA
ICAM-1: intercellular cell adhesion molecule-1
IMCT: intensity-modulated carbon therapy
IMPT: intensity-modulated proton therapy
IMRT: intensity-modulated radiation therapy
LMP: latent membrane proteins
MAPK: mitogen-activated protein kinase
MMP-13: matrix metalloproteinase 13
MTA1: metastatic tumor antigen 1
MVBs: multivesicular bodies
NPC: nasopharyngeal carcinoma
RIG-I: retinoic acid-inducible gene I
PAI: photoacoustic imaging
PD-1: programmed cell death protein 1
PD-L1: programmed cell death-ligand 1
PFKFB-3: enzymes 6-phosphofructo-2-kinase/fructose-2,6-bisphosphatase-3
SDC2: syndecan-2
STAT: signal transducer and activator of transcription
SYTL: synaptophysin-like 4
TGF-β: transforming growth factor-β
TLR3: toll-like receptor 3
Tim-3: T cell immunoglobulin and mucin domain-containing protein 3
Tregs: regulatory T cells
TSGA10: testis-specific gene antigen 10
TSP-1: thrombospondin-1
UCH-L1: ubiquitin carboxyl-terminal hydrolase isozyme L1
VCAM-1: vascular cell adhesion molecule 1
VEGF: vascular endothelial growth factor
